# The Interplay Between Obesity and Type 2 Diabetes: Common Pathophysiological Mechanisms Contributing to Telomere Shortening

**DOI:** 10.3390/life15060873

**Published:** 2025-05-28

**Authors:** Stella Baliou, Miruna-Maria Apetroaei, Eleftheria Hatzidaki, Sergey V. Kuzmin, Manolis N. Tzatzarakis, Andreea Letiția Arsene, Aristides Tsatsakis, Petros Ioannou

**Affiliations:** 1 Laboratory of Toxicology, School of Medicine, University of Crete, 71003 Heraklion, Greece; 2 Faculty of Pharmacy, Carol Davila University of Medicine and Pharmacy, 020956 Bucharest, Romania; 3 Department of Neonatology/NICU, University Hospital of Heraklion, 71110 Heraklion, Greece; 4 School of Medicine, University of Crete, 71003 Heraklion, Greece; 5 Federal Budgetary Establishment of Science “F.F. Erisman Scientific Centre of Hygiene” of the Federal Service for Surveillance on Consumer Rights Protection and Human Wellbeing, 141014 Mytishchi, Russia; 6 Marius Nasta Institute of Pneumology, 050159 Bucharest, Romania

**Keywords:** telomeres, telomere shortening, insulin resistance, oxidative stress, chronic inflammation, type 2 diabetes mellitus, obesity, senescence, aging biomarkers, age-related diseases

## Abstract

The worldwide prevalence of obesity continues to increase, representing a serious public health issue due to associated comorbidities. Obesity is associated with type 2 diabetes mellitus (T2D), which shares similar pathophysiological mechanisms. In both conditions, oxidative stress, inflammation, mitochondrial dysfunction, abnormal adipose tissue function, and senescence are observed, ultimately leading to insulin resistance. In both cases, hypertrophic adipose tissue is associated with telomere shortening. Elucidating the mechanisms underlying telomere shortening in obesity and diabetes may be crucial for deepening our understanding of these pathologies, with the ultimate aim of its translational implications. Several studies have shown that telomere shortening is present in patients with metabolic disorders, emphasizing its prognostic value for the onset and progression of these diseases. From this perspective, this article highlights the importance of telomere biology, which can aid in developing new therapeutic options for metabolic disorders.

## 1. Introduction

According to the World Health Organization, by 2030, one in six people globally will be over 60. This population group is expected to grow from 1 billion (2020) to 1.4 billion (2030) and even 2.1 billion (2050). At the same time, the population over 80 is projected to increase threefold from 2020 to 2050. This phenomenon of the growth of the aging demographic group initially started in developed countries, but it is now on the rise in low- and middle-income countries [1].

Currently, 12 hallmarks of aging are widely used to study the aging process, categorized as primary, antagonistic, and integrative. The main hallmarks include telomere attrition, genetic instability, epigenetic modifications, impaired macroautophagy, and loss of proteostasis. On the other hand, antagonistic hallmarks, which bring little benefit to the organism but can cause damage when their levels are high, are mitochondrial dysfunction, cellular senescence, and unregulated nutrient sensing. Furthermore, the non-reversible combination of the first two categories causes systemic integrative hallmarks such as stem cell depletion, dysbiosis, persistent inflammation, and altered intracellular communication [2,3].

In humans, telomeres represent repetitive hexanucleotide sequences (5′-TTAGGG-3′) extending over several kilobases and concluding in a single-stranded 3′ G-overhang. Telomeric DNA preserves chromosome termini via its association with the hexameric shelterin complex: telomeric repeat-binding factor 1 (TRF1) and telomeric repeat-binding factor 2 (TRF2) establish homodimers and attach to double-stranded DNA, whereas POT1 selectively binds to single-stranded DNA [4]. TIN2 and TPP1 are intermediaries among the DNA-binding proteins, while RAP1 interacts with TRF2. Regarding the shelterin proteins, TRF2 demonstrates a notable ability to encircle DNA and facilitate Holliday junction formation. These distinctive characteristics allow TRF2 to proficiently restructure telomeric DNA into an enclosed t-loop form, wherein the 3′ overhang penetrates the neighboring double-stranded area [5,6]. A critically short telomere length is a major contributor to the onset of replicative senescence in somatic cells and the destabilization of their chromosomes. Among the various molecular changes associated with older age, telomere length has been recognized as one of the best biomarkers of aging [7,8]. However, telomerase is a critical enzyme in sustaining telomere length homeostasis, facilitating the replication of telomeric regions in chromosomal DNA [9].

A lot of research studies have focused attention on understanding the pathophysiology of metabolic disorders, namely obesity and type 2 diabetes (T2D), that are associated with insulin resistance [10]. Interestingly, the most critical determinants of obesity are the increased secretion of non-esterified fatty acids (NEFAs) coupled with hormones and the sustained inflammatory response, thus inducing insulin resistance [11]. When insulin resistance is combined with impaired function of the pancreatic islets and β-cells, T2D emerges [10].

A thorough understanding of the molecular mechanisms that govern telomere dynamics in the context of common metabolic pathologies, such as obesity and diabetes, aims to develop proactive initiatives in scientifically sound preventive strategies. Moreover, decreasing the risk associated with these pathologies and increasing health outcomes, especially considering the inevitable aging characteristics, are at the center of the collective efforts made by health professionals. This narrative review aims to clarify the interconnected molecular pathways in two prevalent pathologies with high mortality rates and to provide solid evidence for assessing telomere dynamics as a diagnostic and prognostic marker and therapeutic target to obtain the necessary information for personalized therapy in these patients.

## 2. Molecular Characteristics in Obesity Pathophysiology

Obesity is a global public health problem, a risk factor for chronic diseases, and a significant economic concern for sanitary authorities [12]. Obesity seems to account for a 39.8% increase in global disability-adjusted life years (DALYs) from 2020 to 2030 [13]. Nowadays, the prevalence of obesity importantly follows a notable increase three times more than that reported in the 1970s [13]. By 2030, obese and overweight individuals will reach 573 million worldwide [14]. Obesity is characterized by excessive fat accumulation in adipose tissue, which harms human health. Increased body fat storage can lead to metabolic disorders, including T2D and cardiovascular disease (CAD), resulting in mortality [15]. The body mass index (BMI), which is a measure of weight (measured in kilograms) divided by size (measured in m^2^), is the primary parameter used to evaluate obesity. According to the World Health Organization (WHO), a value of less than 25 kg/m^2^ is encountered in overweight individuals, whereas a value greater than 30 is observed in obese patients [16]. Even though obesity accelerates the onset of age-related diseases, there is a prominent theory that elevated BMI in elderly patients exerts a protective effect on obese individuals. For example, overweight patients, after coronary artery bypass grafting, developed lower susceptibility to all-cause mortality compared to individuals with normal BMI. It seems that the adiposity unexpectedly confers protection after a stressful event. Conversely, it has been demonstrated that abdominal obesity can aggravate diseases in young individuals, resulting in increased mortality rates associated with abdominal obesity [17].

Adipose tissue is known to be the most vulnerable tissue to age-associated complications, but these changes can be exacerbated in obesity [18]. The adipose tissue is the primary site for worsening obesity-related complications [19,20]. The adipose tissue can be expanded by forming new adipocytes from resident precursors known as preadipocytes (hyperplasia) or increasing the size of existing adipocytes (hypertrophy) [19,20]. In particular, adipose tissue is characterized by impaired cell function since its size, cellular morphology, and distribution have changed. The main characteristics of dysfunctional adipose tissue are represented by senescence, increased fat storage, impaired adipose tissue function and redistribution, reduced ability to respond to insulin, inflammation, oxidative stress, and mitochondrial dysfunction [21].

Numerous studies have revealed the tight relationship between hypertrophic adipose tissue, insulin resistance, and inflammation [22]. Hypertrophic adipose tissue is differently impaired in function, converting it from a metabolic to an immune organ. Low-level chronic inflammation (inflammaging) is crucial in driving insulin resistance [23]. Indeed, immune cells infiltrate the adipose tissue, triggering inflammatory responses, leading to the metabolic shift in the phenotype and function of immune cell subsets [24]. In this context, tissue-resident macrophages have been proposed to induce low-grade inflammation. In hypertrophic adipose tissue, the infiltration of macrophages into adipose tissue is accompanied by increased secretion of pro-inflammatory cytokines, which interfere with insulin signaling [25]. For example, the increased release of tumor necrosis factor-α (TNF-α) and interleukin-6 (IL-6) can exert their action on adipocytes by inhibiting the secretion of adiponectin, interfering with insulin sensitivity [26]. The chronic inflammatory state of the hypertrophic adipose tissue is aggravated through increased secretion of macrophage chemoattractants like monocyte chemoattractant protein-1 (MCP-1), which in turn attracts more macrophages, rendering the insulin-stimulated glucose transport defective, leading to insulin resistance [27]. In response to excess nutrients, adipose tissue becomes hypoxic through increased secretion of the transcription factor hypoxia-inducible factor-1α (HIF-1α). This results in a lack of vascularization, fibrosis induction, and further inflammation [28,29]. In the attempt to elucidate the mechanisms underlying the inflammation-mediated insulin resistance, it has been reported that pro-inflammatory mediators can be triggered by either the nuclear factor-κB (NF-κB) or the c-Jun amino-terminal kinase (JNK) pathway or the protein kinase C (PKC) pathway [30]. The insulin-stimulated glucose transport can be inhibited in muscle through downregulating glucose transporter type-4 (GLUT4), due to the activation of mitogen-activated protein kinase (MAPK) and JNK signaling mediators [27]. As a result, the increased recruitment of immune cells into hypertrophic adipose tissue contributes to low-grade inflammation and the development of insulin resistance [31].

Since senescent cells produce a senescence-associated secreted proteome (SASP) that is composed of pro-inflammatory factors (cytokines, chemokines), extracellular proteases, and growth factors, senescence is considered one of the most critical mechanisms in the hypertrophic adipose tissue, linking obesity and aging [32]. The adipose tissue of obese individuals is characterized by the accumulation of senescent cells to a greater extent than that of lean counterparts [33,34]. In particular, activating the p53 transcription factor in adipose tissue is essential for the aging process of adipose tissue, driving senescence [34]. For example, incubating human preadipocytes with a saturated fatty acid such as palmitic acid leads preadipocytes to senescence, as evidenced by the DNA damage and p16INK4a positive staining in cells [35]. In this context, obesity also causes senescence of all adipose cell types, including mature adipose cells and microvascular endothelial cells [36]. Interestingly, increased recruitment of senescent endothelial cells and pre-adipocytes has been observed in obese individuals compared to their lean counterparts [37]. Comparing the senescent burden of two adipose tissue types, it was substantiated that vascular adipose tissue endothelial cells became more senescent than subcutaneous adipose tissue endothelial cells.

Furthermore, the dysfunctional adipocytes are characterized by reduced lipogenesis and augmented lipolysis. Indeed, the immune infiltration of the adipose tissue causes the increased secretion of pro-inflammatory mediators that account for impaired triglyceride (TG) deposition in the adipose tissue. In particular, TG biosynthesis is reduced through lowering the transactivation capacity of peroxisome proliferator-activated receptor-γ (PPAR-γ), causing reduced TG biosynthesis and storage in the adipose tissue [27]. In obesity, the pronounced senescence of adipose tissue is related to diminished adipogenesis, attributed to a significant reduction in PPARγ expression [38]. As a result, the senescence-mediated reduced adipogenesis is tightly linked to the development of insulin resistance [39] since ectopic lipid accumulation and inflammation are increased in response to excessive caloric intake [39,40].

Furthermore, the defective PPAR-γ function is responsible for lowering levels of lipid-droplet proteins, allowing the further modification of lipid droplets by active lipases and their esterification [27]. In lean adipocytes, activating lipoprotein lipases (LPL) allows TG hydrolysis, enhancing TG biosynthesis and free fatty acid (FFA) esterification into TGs. This process can be potentiated through PPAR-γ, since PPAR-γ is a positive determinant of LPL expression [27]. In contrast, sustainable inflammation compromises fatty acid esterification and induces lipolysis by hindering the action of following PPAR-γ, LPL, and GLUT4 molecules [27]. In this context, the hypertrophic adipocytes show excessive bloodstream FFA circulation and TG release, increasing their deposition in peripheral tissues and impairing insulin signaling [41]. For this reason, obesity is linked to aberrant lipid accumulation in ectopic sites with the accompanying lipotoxicity, insulin resistance, and chronic low-grade inflammation [42], advocating the toxic lipid delivery into the peripheral tissues of obese individuals, such as the heart, skeletal muscle, and pancreas, thereby leading to insulin resistance [43]. For example, 11% and 14% of heart failure cases in men and women, respectively, are caused by obesity-induced cardiac lipotoxicity and concomitant cardiac dysfunction [38].

Similarly, there is substantial proof that obesity disrupts the cellular processes of nutrient sensing. Considering that the mammalian target of rapamycin (mTOR) pathway plays a crucial role in determining the aging process, an increasing body of research has indicated that the mTOR pathway is hyperactivated in obesity, accentuating the aggravation of the aging process [44]. The increased mTOR activity in adipose tissue has been related to developing insulin resistance and hyperlipidemia [45]. Conversely, the downregulated monophosphate-activated protein kinase (AMPK) pathway has been observed in obesity and aging [46,47].

## 3. Molecular Characteristics in Type 2 Diabetes Pathophysiology

Diabetes is a serious health issue worldwide. Due to changes in dietary patterns brought about by urbanization and aging populations, the prevalence of diabetes has increased globally. In 2019, there were more than 463 million people worldwide who had diabetes. By 2045, that number is expected to rise by 46% to 783.2 million people on average [48]. Consequently, diabetes is among the most prevalent health issues affecting all population groups, a varied and complex demographic that encompasses both newly diagnosed older diabetic patients and those with a long history of diabetes that began in middle or early adulthood [49].

The vast majority of diabetic patients fall into one of two broad categories: type 1 diabetes mellitus (T1D), which is caused by an absolute or near-absolute insulin deficiency, or type 2 diabetes mellitus (T2D), which is caused by insulin resistance combined with an insufficient compensatory increase in insulin secretion. Pregnant women who develop diabetes are classified as having gestational diabetes. Moreover, other types of diabetes can be defined, such as diabetes induced by drugs or chemicals, including nicotinic acid, glucocorticoids, thyroid hormone, diazoxide, beta-adrenergic agonists, thiazides, phenytoin, and interferon-alpha [50].

Regarding T2D, it has been reported that it is a prevalent metabolic pathology resulting from either physiologic alterations or their additive effects: insufficient insulin production in β-pancreatic cells and decreased tissue response to insulin activity. The most common risk factors include aberrant elevated blood glucose levels, the presence of other comorbidities (dyslipidemia, obesity), an unhealthy lifestyle (low dietary intake and sedentary lifestyle), physiological aging, genetic predisposition, and other lifestyle factors (stress, anxiety) [51]. Interestingly, the severity of symptoms is directly proportional to the type and duration of diabetes. Polyuria, polydipsia, polyphagia, weight loss, and blurred vision are clinical signs of severe chronic hyperglycemia, which contributes to the progression of uncontrolled diabetes, namely ketoacidosis or hyperosmolar nonketotic coma [52]. These disturbances in metabolism are caused by low insulin levels, insulin resistance in many tissues, mainly skeletal muscle, adipose tissue, and liver, respectively, changes in the signal transduction system, and the effector enzymes or genes in the involved tissues [52]. Interestingly, some studies emphasized that the existence of the disease can influence response to several treatments, as is the case with Direct-Acting Antiviral Agents [53]. Uncontrolled diabetes can lead to coma and, if left untreated, death due to ketoacidosis or nonketotic hyperosmolar syndrome [54]. Long-term complications associated with diabetes fall into two categories: (1) microvascular complications, which include retinopathy, which can lead to vision loss; nephropathy, which can lead to renal failure; and peripheral neuropathy, which can lead to leg ulcerations, amputations, and Charcot joints; and (2) autonomic neuropathy, which may manifest as gastrointestinal, genitourinary, and cardiovascular symptoms and sexual dysfunction [55].

**Figure 1:**
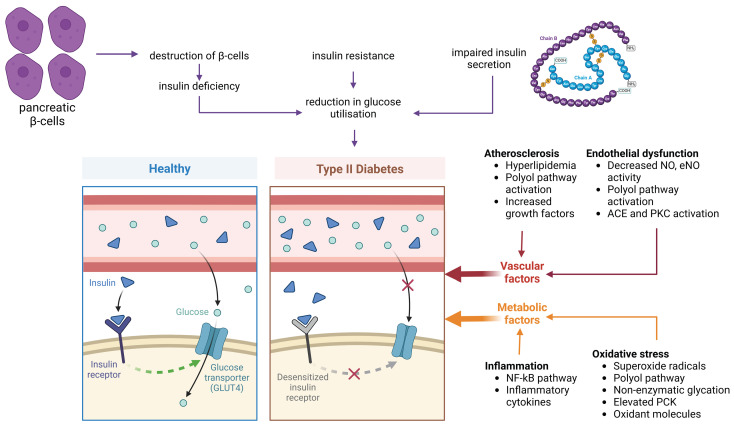
Pathophysiological alterations in type 2 diabetes (T2D). Insulin resistance results from β-cell dysfunction, impaired insulin secretion, and reduced glucose uptake due to desensitized insulin receptors. In healthy conditions, insulin binds to its receptor, facilitating glucose entry into cells via glucose transporter type 4 (GLUT4). In T2D, this signaling is impaired. Contributing vascular factors include atherosclerosis (triggered by hyperlipidemia, activation of the polyol pathway, and increased growth factors) and endothelial dysfunction (characterized by reduced nitric oxide (NO) and endothelial nitric oxide synthase (eNOS) activity, as well as activation of angiotensin-converting enzyme (ACE) and protein kinase C (PKC)). Metabolic factors involve chronic inflammation (mediated by nuclear factor kappa B (NF-κB) and inflammatory cytokines) and oxidative stress (driven by superoxide radicals, polyol pathway activity, non-enzymatic glycation, elevated protein kinase C (PKC), and excess oxidant molecules) (created with Biorender.com).

Since hyperglycemia is the main clinical symptom of diabetic disease, numerous important mechanisms underlying the vascular damage caused by hyperglycemia have been analyzed. The primary reasons for hyperglycemia in T2D are the insufficient ability to absorb glucose, hormonal disturbance of glucose metabolism, and abnormal pancreatic β-cell secretory activity (Figure 1). Due to hyperglycemia, the biological function of numerous organs and the multitude of metabolic processes are negatively affected [56].

**Figure 2:**
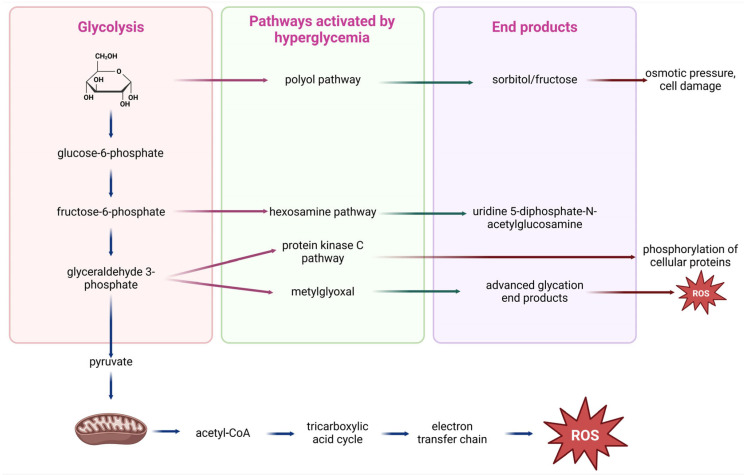
Hyperglycemia-activated signaling pathways. In conditions of high blood glucose, glycolysis intermediates such as glucose-6-phosphate, fructose-6-phosphate, and glyceraldehyde 3-phosphate divert into alternative metabolic routes. These include the polyol pathway (producing sorbitol and fructose, leading to osmotic pressure and cellular damage), the hexosamine pathway (producing uridine 5-diphosphate-N-acetylglucosamine (UDP-GlcNAc), which promotes abnormal phosphorylation of cellular proteins), and the protein kinase C (PKC) pathway, which disrupts cellular signaling. Additionally, methylglyoxal formation leads to the accumulation of advanced glycosylation end-products (AGEPs), contributing to oxidative stress. All pathways increase reactive oxygen species (ROS), which are also generated in mitochondria through the tricarboxylic acid (TCA) cycle and electron transport chain (ETC) after conversion of pyruvate to acetyl coenzyme A (acetyl-CoA), further amplifying oxidative stress and cellular dysfunction (created with Biorender.com).

From a molecular perspective, the upregulation of PKC, polyol or hexosamine pathway and the increased secretion of advanced glycation end-products (AGE) or growth factors (GFs) are the underlying mechanisms of hyperglycemia that mediate oxidative stress (Figure 2) [57]. Then, the reactive oxygen species (ROS) accumulation and the downregulation of pro-oxidant molecules create a negative feedback loop that exacerbates diabetic vascular complications [58]. For example, higher intracellular glucose concentrations trigger the activation of the PKC pathway, which in turn induces the generation of ROS, nicotinamide adenine dinucleotide phosphate (NADPH) oxidase and the p66Shc adaptor protein [59].

Additionally, it is crucial to emphasize the interplay between oxidative stress and endothelial dysfunction. Due to hyperglycemia, the upregulation of the PKC pathway, in turn, leads to an imbalance in endothelial nitric oxide synthase (eNOS), thereby perpetuating oxidative stress. In this regard, hyperglycemia inhibits eNOS phosphorylation at Ser1177, thus compromising eNOS activity. Indeed, reduced NO and elevated PKC activity exert overlapping synergistic effects, converging on the induction of endothelin-1 (ET-1), which is crucial for platelet aggregation and vasoconstriction [60]. Moreover, T2D is associated with endothelial dysfunction, characterized by increased amounts of thromboxane A2 (TXA2) resulting from the overexpression of cyclooxygenase-2 (COX-2) and the inhibition of prostacyclin synthetase. This mechanism is triggered by an aberrant nitrosylation reaction [61].

The activation of the immune system is also associated with the onset and development of T2D; both adaptive and innate immune responses contribute to adipose tissue inflammation. The transition of macrophages from primarily anti-inflammatory M2-type to a higher proportion of pro-inflammatory M1-type macrophages is responsible for initiating and intensifying inflammation in the islets. Notably, emerging evidence suggests that the recruitment of B and T cells precedes the infiltration of macrophages into adipose tissue [62].

## 4. Linking the Common Molecular Pathways Between Obesity and Diabetes

The term “diabesity” is employed to point out the close relationship between obesity and diabetes, precisely the fact that the majority of patients with T2D are overweight or obese [63]. Obesity is often accompanied by T2D, which is non-insulin-dependent diabetes. Kahn and Flier highlighted that obesity increases the susceptibility to T2D occurrence [64]. By 2025, it is estimated that the prevalence of these two comorbidities will increase, reaching 300 million individuals having both obesity and T2D [65]. As anticipated, the two disorders cannot be separated but should be studied as a single disease. The relationship between these conditions acquires more importance since mounting evidence shows that obesity is the major contributory factor for T2D [64]. Previous research showed that 85.2% of T2D patients were obese [66].

The significant characteristics of T2D are reduced insulin generation and disturbed insulin secretion, which contribute to insulin resistance [63]. Moreover, obesity can aggravate insulin resistance since it is a risk factor for T2D development [67]. For example, obese people without T2D usually have higher plasma levels of insulin and secrete more insulin than lean individuals, whether consuming glucose or not [10]. It is also well known that as people age, their insulin secretion declines [68]. In addition to the capacity of pancreatic β-cells to secrete insulin, the β-cell mass determines the levels of secreted insulin [69,70].

**Figure 3:**
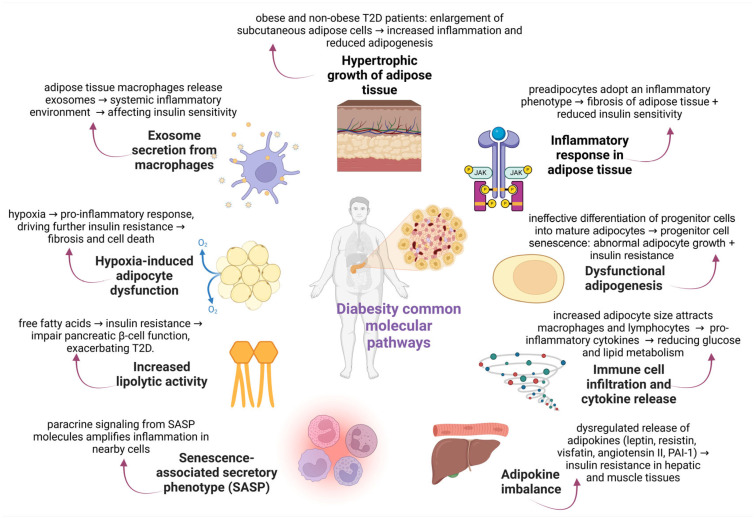
The molecular mechanisms that link obesity and type 2 diabetes (T2D). Enlarged fat cells in obesity contribute to hypertrophic growth of adipose tissue, promoting inflammation and impairing adipogenesis. Inflammatory responses in adipose tissue arise as preadipocytes adopt pro-inflammatory phenotypes, reducing insulin sensitivity. Impaired differentiation of progenitor cells results in dysfunctional adipogenesis, contributing to insulin resistance. Immune cell infiltration and cytokine release occur as hypertrophic adipocytes attract macrophages and lymphocytes, releasing pro-inflammatory cytokines that disrupt glucose and lipid metabolism. The dysregulation of adipose-derived hormones, or adipokine imbalance [e.g., leptin, resistin, visfatin, angiotensin II, plasminogen activator inhibitor-1 (PAI-1)], promotes insulin resistance in the liver and muscle. Senescence-associated secretory phenotype (SASP) factors amplify inflammation through paracrine signaling. Increased lipolytic activity raises free fatty acids, impairing pancreatic β-cell function. Hypoxia-induced adipocyte dysfunction drives inflammation, fibrosis, and cell death. Exosome secretion from macrophages enhances systemic inflammation, exacerbating insulin resistance and contributing to the pathophysiology of diabesity (created with Biorender.com).

Research has revealed several intricate, interconnected molecular mechanisms in adipose tissue that support insulin resistance in obesity and T2D (Figure 3). Fatty tissue hypertrophy is considered the main characteristic of obesity, linking this condition with T2D and eventually leading to insulin resistance [71]. Abnormal hypertrophic cell growth in subcutaneous adipose tissue has also been described in non-obese persons with T2D [46] and non-diabetic individuals with a genetic predisposition for T2D (first-degree relatives; FDR), regardless of age [71].

From a molecular perspective, the primary mechanism underlying insulin resistance in people with obesity and T2D is the hypertrophic growth of subcutaneous adipose cells in humans, related to increased local and systemic inflammation and reduced adipogenesis [31,72,73,74]. First, dysfunctional adipogenesis is the ineffective differentiation of adipose progenitor cells into new mature adipose cells [31]. Indeed, the increased adipose progenitor cell senescence contributes to hypertrophic adipose cell growth related to increased insulin resistance and dyslipidemia [39]. Secondly, preadipocytes may adopt an inflammatory phenotype in these conditions, exhibiting diminished adipogenic capacity and increased expression of pro-inflammatory cytokines, ultimately resulting in adipose tissue fibrosis [75]. In hypertrophic adipose tissue, dysfunctional adipocytes release high amounts of growth hormones, extracellular matrix proteases, and inflammatory molecules comprising senescence-associated secretory phenotype (SASP) that exert their effect on neighboring cells in a paracrine manner [76]. Indeed, the adipose tissues of obese mice have been illustrated to exhibit an upregulation of macrophage-specific genes, such as MCP-1, macrophage inflammatory protein-1α (MIP-1α), CD68, CD11b, and F4/80 [77]. Adipocyte size and the percentage of F4/80 expression were significantly correlated by immunohistopathological assays in the adipose tissue [77]. In this manner, increased adipocyte size has been reported to positively correlate with increased macrophage infiltration into the fatty tissue [77]. As a result, the risk of acquiring insulin resistance and T2D is higher in individuals with elevated circulating levels of pro-inflammatory mediators (CRP, IL-6, and IL-1β) and in obese individuals [22,37,78,79]. For this reason, the increased recruitment of the following transcription factors NF-κB, CCAAT/enhancer-binding protein-β (C/EBP-β), and GATA4 to the promoter regions of SASP genes has been observed, increasing the SASP gene expression at the transcriptional level [80].

Regarding the association of inflammation and lipid metabolism in hypertrophic adipose tissue, it has been reported that the increased release of pro-inflammatory cytokines from immune cells impairs the insulin signaling pathway, ending in dysregulated metabolism of glucose and fatty acids and reduced insulin sensitivity [81,82,83]. In particular, the elevated lipolytic activity in adipose tissue and the increased secretion of FFAs into the bloodstream contribute to insulin resistance [84]. Non-esterified fatty acids are essential for the onset of insulin resistance and pancreatic β-cell function impairment in obese people [64]. In this regard, adipose tissue’s hypoxic response facilitates the death of adipocyte cells and fibrosis, proceeding with the induction of a pro-inflammatory response. In hypertrophic adipose tissue, the local hypoxia activates multiple adipokines’ secretion and boosts immune cells’ infiltration, mainly monocytes and T and B lymphocytes [77]. In this regard, adipocyte tissue dysfunction causes ectopic lipid deposition in other peripheral tissues, including the liver and muscle [85]. As a result, the persistent inflammatory response can cause ectopic lipid accumulation in the bloodstream, ultimately leading to insulin resistance.

Since adipokines exert pro-inflammatory or anti-inflammatory functions in a hormone-like manner, significant progress has been made in understanding the role of adipokines in metabolic disorders [86]. In this direction, dysregulated adipocytes have been illustrated to release higher levels of inflammatory cytokines (IL-6 and IL-1β) and adipokines, including leptin, visfatin, resistin, angiotensin II, and plasminogen activator inhibitor-1 (PAI-1) [87], thus contributing to a low-grade inflammatory state and insulin resistance [86,88]. In another case, hepatic and skeletal muscle insulin sensitivity are inversely linked with plasma PAI-1 levels, which are more significant in the obese insulin-resistant group [89]. In contrast, the reduced expression levels of anti-inflammatory adipokines are a vital hallmark that characterizes obesity [90]. For example, the increased recruitment of immune cell populations in hypertrophic adipose tissue prevents adiponectin release [91].

Accordingly, there is a wealth of information that the aging process and obesity can disrupt stem cell renewal. During aging, the ability of stem cells to regenerate themselves worsens, and their ability to differentiate into different cell types declines [92]. In the meantime, obesity does not mimic aging, but obesity is also considered detrimental to stem cell function, aggravating this phenomenon [92].

To sum up, high rates of fibrogenesis, increased levels of proinflammatory T cells and macrophages, the expression of genes that encode proinflammatory mediators, and the great generation of exosomes are significant drivers of insulin resistance [93,94]. All the parameters mentioned earlier contribute to insulin resistance. In adults, reduced leukocyte telomere length correlates with metabolic diseases, including obesity and type 2 diabetes mellitus. These correlations may originate from early childhood interactions between telomere length and metabolic diseases. Short telomeres facilitate the onset of obesity and metabolic diseases at an early age, significantly affecting health [95].

## 5. Obesity and Telomere Length

### 5.1. Molecular Mechanisms Underlying Telomere Shortening in Obesity

In obesity, insulin resistance develops in adipose and peripheral tissues due to oxidative stress and an increased inflammatory response [96,97,98]. Lipid peroxidation is also associated with insulin resistance [99]. On the one hand, oxidative stress facilitates the onset and advancement of metabolic dysfunction by generating a pro-inflammatory milieu or exerting its influence on insulin receptors, thereby contributing to peripheral insulin resistance [100,101]. In this direction, a systematic, comprehensive review of studies has provided convincing evidence that oxidative stress induces a pro-inflammatory state [102]. On the other hand, inflammation can drive oxidative stress. Since adipocytes and preadipocytes are identified as reservoirs for proinflammatory cytokines, including TNF-α, IL-1, and IL-6, obesity is a chronic inflammatory condition. These pro-inflammatory mediators are possible activators of the synthesis of reactive oxygen species (ROS) and reactive nitrogen species (RNS) by macrophages and monocytes. For example, TNF-α increases the chemical reaction between electrons and oxygen, increasing superoxide anion (O_2_^−^) levels [103].

Obesity is also associated with changes in the mitochondrial metabolism of adipocytes. In conditions of increased adiposity, adipocytes exhibit impaired mitochondrial respiratory capacity and diminished mitochondrial biogenesis [104]. On the one hand, defective mitochondria generate reduced adenosine triphosphate (ATP) and increased reactive oxygen species (ROS) production due to insufficient activity in the mitochondrial electron transport chain complexes [105]. In this perspective, the mitochondrial process of oxidative phosphorylation is less efficient, inducing the generation of harmful ROS and compromising the synthesis of ATP [106]. Alternatively, mitochondrial oxidation of fatty acids comprises another route for synthesizing ROS during obesity [41]. In this case, peroxisomes can oxidize fatty acids in this pathology, generating ROS accumulation and creating oxidative stress [41]. On the other hand, decreased mitochondrial biogenesis can cause metabolic changes, low-grade inflammation, and insulin resistance [107]. Another parameter involved in inhibiting mitochondrial turnover is the prevention of mitophagy, a central process for removing dysfunctional mitochondria [108]. In particular, the mammalian target of rapamycin (mTORC1) mediates the phosphorylation at the following targets–unc-51-like kinase 1/2 (ULK-1 and ULK-2)–in their inhibitory sites, thereby counteracting mitophagy [109].

To understand the effect of oxidative stress on telomere length homeostasis in hypertrophic adipose tissue, the research has focused on elucidating the link between oxidative stress and telomere shortening in obese people and its underlying molecular mechanisms [110]. In Caucasian men, hypertension and insulin resistance exert detrimental effects on telomere length values in their leukocytes [111]. Accordingly, obesity is associated with elevated lipid peroxidation levels in plasma, as indicated by an increase in 8-epi-prostaglandin F2α (8-epi-PGF2α) [112]. Pursuing this further, an inverse relationship is observed between high 8-epi-PGF2α levels and leukocyte telomere length [111]. This finding suggests that oxidative stress in hypertrophic adipose tissue may be a crucial driving force in accelerating the aging process by impairing telomere length values [113,114]. From a molecular perspective, the oxidative stress in adipose tissue contributes to senescence and telomere shortening due to DNA damage, aggravating the aging phenotype [115,116]. Alternatively, oxidative stress can affect the gene expression of the shelterin complex, thereby regulating telomere length values [117]. Interestingly, obese people with oxidative stress and increased expression of negative regulators of the shelterin protein complex present accelerated telomere shortening rates [118]. Based on this, TRF1 has been identified as a significant protein in the shelterin protein complex, negatively affecting telomere length. In particular, principal component analysis has proved that the upregulation of TRF1 seems to drive increased rates of telomere shortening [118].

Regarding the effect of inflammation on telomere length values in obesity, the increase in BMI seems to trigger the increased secretion of inflammatory mediators, including fibrinogen, C-reactive protein (CRP), IL-6, and TNF-α, which in turn cause accelerated telomere shortening [98,119]. These results align with other findings, which indicate that high consumption of omega-6 polyunsaturated fatty acids (n-6 PUFAs) with pro-inflammatory properties is associated with telomere shortening [120].

In the molecular setting, several researchers have elucidated the molecular mechanisms underlying inflammation’s negative impact on telomere length homeostasis. Interestingly, the proinflammatory [121] macrophages secrete high levels of proinflammatory mediators like TNF-α and IL-6 through NF-κB transcriptional upregulation, leading to increased telomere shortening rates [122]. Similarly, the activation of NF-κB pro-inflammatory signaling by TNF-α is associated with higher levels and activation of the telomerase [123]. In line with this, the non-phosphorylated NF-κB-p65 has been observed to accumulate in the nucleus when telomerase is overexpressed. The nuclear transition of the non-phosphorylated NF-κB p65 subunit does not influence NF-κB-p65 signaling but can increase protein stability by preventing its degradation through the ubiquitin–proteasome system [124,125].

In addition, hypertrophic adipose tissue presents characteristics of endothelial dysfunction that, in turn, modulate telomere length homeostasis. In increased adiposity conditions, high levels of superoxide radicals and nitrotyrosine in the endothelium and high levels of pro-inflammatory mediators seem to cause endothelial dysfunction [126]. The hallmarks of endothelial dysfunction are the reduced bioavailability of blood vessel dilation agents, especially nitric oxide (NO), and increased endothelium-derived contractile factors. Endothelial impairment is also evidenced by exerting pro-inflammatory, proliferative, and procoagulant properties [102]. Combining and amplifying such modifications can contribute to metabolic and vascular changes [103,127]. In the study by Satoh et al., endothelial progenitor cells (EPCs) from patients with coronary artery disease (CAD) had a significant decrease in telomere length values, which were linked to oxidative DNA damage and endothelial dysfunction through impaired nitric oxide (NO) bioavailability [128]. The telomere shortening was even more pronounced in CAD patients with metabolic syndrome, entailing the critical role of endothelial cell dysfunction in aggravating senescence by adversely affecting telomere length values [128]. In line with this, endothelial dysfunction can drive increased rates of telomere shortening through oxidative stress, as proved in patients with metabolic syndrome [129]. In this context, it has been reported that telomere dysfunction can serve as a mechanism behind endothelial cell dysfunction [130]. In particular, reduced expression levels of shelterin TRF2 protein can lead to senescence of endothelial cells through inflammation and oxidative stress, contributing to the emergence of metabolic and vascular complications [130]. In contrast, MacEneaney showed that increased adiposity was associated with EPC dysfunction without affecting further telomere shortening. Interestingly, the compromised EPC reparative potential was linked to increased adiposity without diminishing further telomere length values [131]. When APCs were disturbed, they did not release angiogenic cytokines, rendering EPCs more susceptible to apoptosis and lowering their cell migration capacity without causing a further decrease in telomere length values in increased adiposity [131].

**Figure 4:**
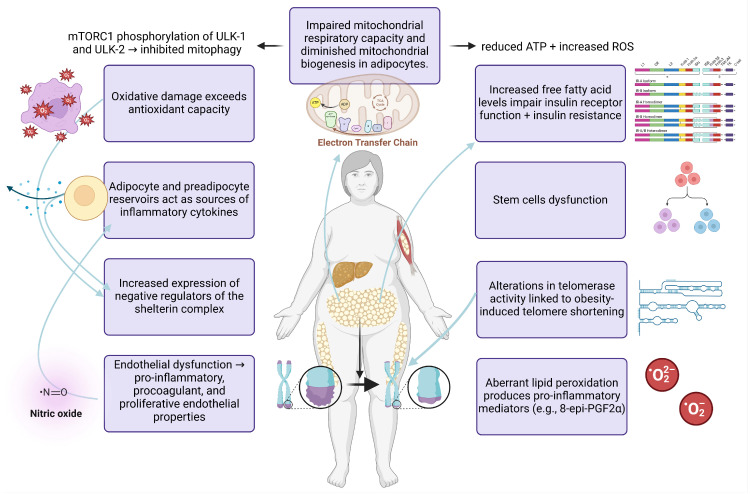
Molecular mechanisms underlying telomere shortening in obesity. Obesity disrupts mitochondrial function in adipocytes, leading to impaired mitochondrial respiratory capacity, reduced adenosine triphosphate (ATP) production, and elevated reactive oxygen species (ROS). Activation of mechanistic target of rapamycin complex 1 (mTORC1) inhibits mitophagy by phosphorylating unc-51-like kinase 1 and 2 (ULK-1, ULK-2). Excess oxidative damage surpasses the antioxidant response, promoting chronic inflammation as adipocytes and preadipocytes release cytokines. Elevated free fatty acids impair insulin receptor signaling and promote insulin resistance. Endothelial dysfunction, marked by reduced nitric oxide (NO), fosters a pro-inflammatory and pro-coagulant environment. Obesity also impairs stem cell function and increases expression of negative regulators of the shelterin complex, destabilizing telomeres. Altered telomerase activity and aberrant lipid peroxidation (e.g., the production of 8-epi-prostaglandin F2α (8-epi-PGF2α)) further accelerate telomere shortening and cellular aging (created with Biorender.com).

To sum up, increased inflammatory response, oxidative stress-related reduced mitochondrial capacity, endothelial dysfunction, free fatty acid overabundance, stem cell disturbance, increased expression of inhibitors of telomere binding factors like shelterin protein complex and altered telomerase action are the mechanisms underlying telomere shortening in increased adiposity conditions (Figure 4). The increased inflammatory response and oxidative burst are two important underlying mechanisms of increased adiposity, which leads to accelerated telomere shortening through DNA damage accumulation [132]. Indeed, the increased response of cells to damaged DNA induces the activation of the p53 transcription factor, eventually leading to senescence [115]. As a result, inflammation and oxidative stress drive irreversible DNA damage, eventually leading to senescence and telomere shortening.

In this context, some therapeutic interventions seem to alleviate the detrimental effects of increased adiposity on telomere length values by attenuating oxidative stress and inflammation. Such options prevent telomere shortening, thus helping to manage metabolic disorders. The high intake of antioxidant substances in the Mediterranean diet has been proven to protect telomeres against oxidative damage [133]. Polyphenols, minerals, vitamins, and the reduced ratio of dietary omega-6–omega-3 fatty acids can sustain telomere length, alleviating oxidative overload [133]. The Mediterranean diet is also enriched in monounsaturated fatty acids, like those from olive oil, which can prevent telomere shortening in oxidative stress conditions [133]. In addition, other modalities like physical activity alleviate the redox state, leading to telomere length maintenance. Like antioxidants, other anti-inflammatory factors contribute to telomere length maintenance. Particular emphasis has been placed on resveratrol, statins, curcuminoids, and catechins, which alleviate the inflammatory response by regulating telomerase activity [133,134].

Last, bariatric surgery remains the most effective way to deal with obesity [135]. Initially, a ten-year prospective study showed that bariatric patients showed telomere length elongation, providing evidence about the beneficial effect of weight loss on telomere length homeostasis [136]. Accordingly, a recent meta-analysis has offered convincing evidence about the beneficial effect of bariatric surgery on telomere length elongation [137]. The mechanisms underlying the favorable impact of bariatric surgery involve facilitating oxidative and inflammatory responses in obese individuals.

### 5.2. Obesity and Telomere Length in Empirical Studies

It is well established that obesity is closely associated with accelerated telomere shortening. A growing body of evidence has shown that obese individuals are characterized by shorter telomeres than eutrophic individuals [138,139]. Most studies have used BMI as a marker for monitoring increased adiposity. In this direction, a systematic review has highlighted that high BMI is associated with insulin resistance due to high telomere shortening rates [140]. This phenomenon of telomere shortening is more pronounced in young obese individuals, excluding the negative effect of aging, and implying the causal relationship of BMI with telomere shortening [140]. In another study, young adults of the Bogalusa Heart Study exhibit changes in their insulin resistance according to their telomere length values in the time region of 10.1 to 12.8 years, suggesting a tight link between telomere shortening and insulin resistance [141]. Likewise, the increased BMI of obese people seems to reduce their telomere length by 16% [142]. In this study, the subcutaneous adipose tissue cells of obese people present higher telomere shortening rates than those of the matched controls after adjusting for age, triglyceride, fasting glucose, and smoking status [142]. Accordingly, individuals, especially women with higher abdominal adiposity and greater BMI, are characterized by telomere shortening [143]. It is also important to note that the inverse relationship of BMI with telomere length has been established, regardless of sex, age, insulin and blood glucose levels, cholesterol and lipoprotein concentrations, level of regular exercise, smoking status, or other metabolic changes. In this perspective, telomere shortening seems to depend on high BMI and waist-to-hip ratio [144].

In line with the above, several meta-analyses have highlighted the negative effect of obesity on telomere length values, taking into consideration different parameters. In a systematic review composed of 63 studies that recruited 119,439 individuals, the negative association between obesity and telomere length is substantiated, considering confounders such as age, telomere length measurement method, tissue type (adipose tissue or blood), and country of origin [145]. This systematic review provides convincing evidence advocating the causal role of obesity in mediating telomere shortening [145]. The major limitation of this meta-analysis is its high heterogeneity, since different methods are used for evaluating telomere length in populations [145]. Despite the high heterogeneity of included studies, the systematic review provides essential clues about the inverse correlation of obesity and telomere length, regardless of the health status, race, gender, and age of participants [145]. Accordingly, another meta-analysis composed of both cross-sectional and longitudinal studies has illustrated that telomere shortening is accelerated in a BMI-dependent manner. However, that meta-analysis cannot provide conclusive results since it analyzes participants’ health state and telomere length without considering their age and sex [146]. In line with this, another meta-analysis has provided deep insights into this inverse relationship, underscoring that each unit of BMI increase corresponds to a respective decrease of 3.99 base pairs (bps) in telomere length, with the results being more apparent in younger individuals (18–60 years) [147]. This decline is more evident in young adults since the telomere length was reduced by 7.67 bps with each BMI unit increase [147]. The telomere length values did not differ, adjusting for the age and sex of individuals in the population [147]. However, it is worth noting that increased BMI can positively affect patients with other comorbidities. For example, colorectal cancer patients with obesity delay their rate of telomere shortening compared to those with an average BMI [148].

Apart from BMI, different parameters of increased adiposity, including waist circumference, waist-to-hip ratio, and total fat, all demonstrate a negative association with telomere length [144,149,150]. Even after adjusting for age, Njajou et al. and García-Calzón et al. have revealed an essential relationship between telomere shortening and greater waist circumference [151,152]. In this context, the MRC National Survey of Health and Development showed a weak inverse relationship among BMI, waist and hip circumference, and leukocyte telomere length [153]. Combining different factors of obesity, it has been proved that BMI, weight, hip circumference, waist-to-height ratio, and waist circumference are negatively correlated with telomere length; however, such associations are not observed with the waist-to-hip ratio. For example, women above the age of 55 who have not gained weight present telomere length elongation compared to those who are obese or overweight [140].

In addition, nuchal subcutaneous adipose tissue thickness and carotid intima-media thickness (IMT) are generally employed to evaluate obesity. Interestingly, the STYJOBS/EDECTA study (STYrian Juvenile Obesity Study, Early DEteCTion of atherosclerosis) has provided information that obesity is inversely related to telomere length homeostasis [146]. In particular, obese individuals have impaired metabolic profiles, as evidenced by nuchal subcutaneous adipose tissue thickness, IMT, high BMI, and hip and waist circumferences, contributing to an increased rate of telomere shortening [146]. In another case, obese individuals present higher telomere shortening rates in their subcutaneous adipose tissue depots than others, implying the higher susceptibility of subcutaneous adipose tissue to aging-related complications [154]. Consistent with the above, the negative association of obesity parameters with telomere length has been reported from the results of the Malmö Diet and Cancer Cohort (MDCC) and the Northern Sweden MONICA project [150].

Regarding the telomere length changes in the adipose tissue, it has been reported that telomere shortening can be accomplished to a greater extent in subcutaneous adipose tissue compared to visceral adipose tissue [154].

Even though several extensive studies have proved the tight relationship between obesity and telomere shortening, some studies advocate the nonsignificant associations between telomere length and obesity markers [155]. Table 1 summarizes relevant murine models that link obesity and its effect on telomere dynamics.

## 6. Type 2 Diabetes (T2D) and Telomere Length

### 6.1. Molecular Mechanisms Underlying Telomere Shortening in T2D

Early prognosis of health conditions for individuals with diabetes mellitus is a valuable strategy to mitigate associated chronic problems and the risk of premature mortality. Telomere length is affected by both hereditary and environmental variables, with shorter telomeres linked to and predictive of negative cardiometabolic outcomes [158].

First and foremost, diabetes seems to contribute to telomere shortening through the induction of oxidative stress [159]. There is substantial empirical evidence for increased oxidative stress and telomere shortening in diabetes mellitus [110,160]. Indeed, the exact mechanisms underlying the negative link of telomere shortening with hyperglycemia are attributed to an imbalance of oxidants and pro-oxidants [161]. In diabetic conditions, increased triglyceride, total cholesterol, and low-density lipoprotein (LDL) cholesterol levels are associated with elevated malondialdehyde (MDA) and compromised activity of key antioxidant enzymes such as glutathione peroxidases and superoxide dismutase in plasma [162]. The excessive ROS accumulation contributes to DNA mutations and destabilization of electron transport complexes, leading to senescence and cell apoptosis. In simple terms, excessive ROS accumulation destabilizes mitochondrial processes, leading to mitochondrial DNA mutations and the erosion of essential macromolecules, as well as the disruption of the NADPH balance [163].

**Figure 5:**
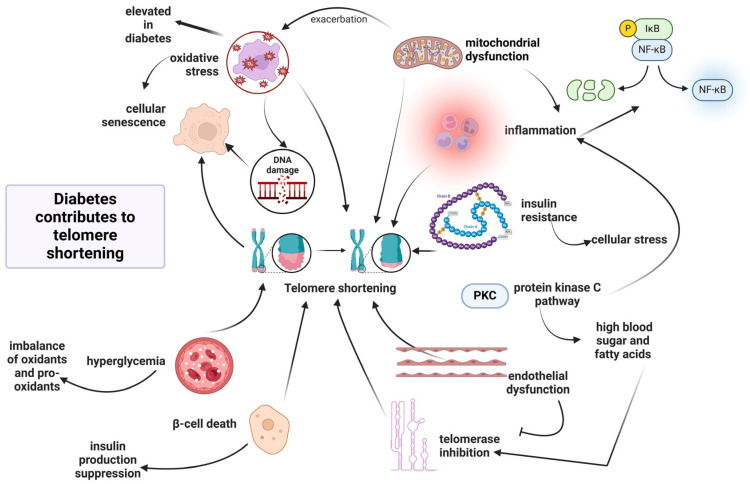
Molecular interplay between T2D and telomere dynamics. Legend: Diabetes contributes to telomere shortening through multiple interconnected mechanisms. Elevated oxidative stress, common in T2D, induces DNA damage and promotes cellular senescence. Mitochondrial dysfunction further exacerbates ROS production, while chronic inflammation, mediated by nuclear factor kappa B (NF-κB), amplifies cellular stress. Insulin resistance driven by high blood glucose and fatty acids activates the protein kinase C (PKC) pathway, contributing to endothelial dysfunction and telomerase inhibition. Hyperglycemia causes an imbalance between oxidants and antioxidants, leading to β-cell death and impaired insulin production. Collectively, these alterations trigger progressive telomere shortening, impairing genomic stability and accelerating biological aging in individuals with T2D (created with Biorender.com).

Secondly, telomere shortening can exacerbate T2D pathology by inducing β-pancreatic cell death, inhibiting insulin production [164]. The pancreatic β-cells play a crucial role in this disorder, being susceptible to oxidative stress due to their efforts to maintain insulin production [165]. In this context, a negative association has also been observed between monocyte telomere length and oxidative stress-mediated DNA damage in monocyte precursors derived from patients with T2D [113]. Accordingly, T2D patients are susceptible to a higher rate of telomere shortening due to elevated oxidative stress conditions [114]. Interestingly, T2D patients with low antioxidant response and genetic UCP2 variants exhibit an accelerated telomere shortening rate, suggesting that the inverse association between telomere length values and T2D is due to excessive ROS generation [114]. These results suggest that UCP2 variants contribute to excessive ROS production, which in turn causes telomere damage [114]. Figure 5 illustrates the complex interplay of molecular mechanisms contributing to telomere length shortening in T2D.

Third, high blood sugar levels, aberrant fatty acid release, and inadequate nutritional intake contribute to increased oxidative status at the tissue level in the long term, thereby affecting the insulin signaling pathway and stimulating the release of proinflammatory cytokines, including TNF-α, transforming growth factor-β (TGF-β), and NF-κB [57,166]. Indeed, pro-inflammatory cytokines are in higher concentrations in diabetic patients than in healthy individuals [160]. Additionally, chronic inflammation accelerates the shortening of telomeres, further aggravating the symptoms associated with diabetes. Furthermore, patients with impaired glucose tolerance have statistically significantly shorter telomeres due to insulin resistance. This telomere shortening occurs due to compromised preservation mechanisms and cellular stress triggered by insulin resistance [167]. Inflammation can induce excessive oxidative stress, reducing telomere length [168]. In the molecular setting, cyclooxygenase-2 (COX-2) is a critical enzyme activated by NF-κB in the peripheral nerves of patients with diabetes and leads to the formation of prostaglandin E_2_ (PGE_2_) and ROS, which further activate NF-κB pathway in a vicious cycle [169]. Consequently, persistent activation of NF-κB is central to all inflammatory processes linked to diabetes [170]. Pro-growth and survival pathways that foster aging phenotypes, such as insulin/insulin-like growth factor 1 (IGF-1) signaling, are also triggered through the activation of the NF-κB pathway [171]. Precisely, insulin/IGF-1 activates PI-3K/AKT, which subsequently stimulates NF-κB, while also activating mTOR, a mediator of stress response that functions as a pro-aging factor, activating NF-κB. Additionally, inflammation, stress pathways, and DNA damage contribute to age-related changes via mechanisms that culminate in the activation of NF-κB signaling. Telomere shortening leads to genomic instability and cellular senescence, promoting aging by activating NF-κB and elevating the levels of COX-2 and ROS. In turn, NF-κB positively impacts the expression of the catalytic subunit TERT, further contributing to telomere shortening. The interaction between TERT and NF-κB creates a feedback loop, where NF-κB signaling enhances macrophage polarization to an inflammatory M1 phenotype, leading to elevated levels of IL-6 and TNF-α, thereby exacerbating inflammation [172,173,174].

In T2D pathology, inflammation and oxidative stress are the most important characteristics, engaging the human body in a continuous pro-inflammatory and pro-oxidative state in a vicious cycle, rendering cells more susceptible to DNA damage, senescence, and insulin resistance. All of these metabolic pathways activated by chronic hyperglycemia contribute to marked oxidative stress, which induces double-strand breaks (DSBs) in the DNA and subsequently leads to the activation of ataxia telangiectasia mutated (ATM) and checkpoint kinase 2 (CHK2), which activate the p53 signaling pathway and induce senescence [175]. Activated p53 increases p21 expression, inhibiting cyclin-dependent kinase 2 (CDK2) from phosphorylating retinoblastoma and causing cell cycle arrest at the G1/S checkpoint [175].

In the molecular setting, pro-inflammatory stimuli can concurrently trigger the c-Jun amino-terminal kinase (JNK) and inhibitory kappa B kinase beta (IKKβ) pathways. The activation of JNK and IKKβ/NF-κB is induced by pro-inflammatory cytokines, such as TNF-α and IL-1β, through receptor-mediated pathways. Additionally, non-receptor mechanisms are also involved, mainly through the recognition patterns of glycation end-product receptor (RAGE) surface proteins that identify foreign substances. JNK promotes insulin resistance by phosphorylating serine residues in insulin receptor substrate-1, and IKKβ contributes to insulin resistance through the transcriptional expression of NF-κB. When the JNK and NF-κB pathways are activated, pro-inflammatory cytokines and mediators produce additional stimulation through feed-forward mechanisms, exacerbating all cell signaling pathways that contribute to telomere shortening [176,177]. These molecular alterations induce senescence indicators such as phosphorylated H2AX (a biomarker of DNA damage) and increased p53 expression, accelerating cellular aging [178].

In more detail, hyperglycemia contributes to telomere shortening through the activation of the polyol pathway, the advanced glycosylation end-products (AGEPs) pathway, the protein kinase C (PKC) pathway, or the hexosamine pathway, all of which induce conditions of oxidative stress and inflammation. Processes in the AGEPs pathway and the polyol pathway alter the cell’s redox capacity by depleting components essential for glutathione recycling or directly producing reactive oxygen species [179]. On the other hand, the hexosamine pathway and PKC activation can trigger the generation of inflammatory factors [169].

Regarding the contribution of the polyol pathway to telomere shortening, this pathway is the main contributor to the NADH/NAD^+^ redox imbalance in diabetes since nearly 30% of blood glucose can be converted through it [180]. An example of the implication of the polyol pathway in generating oxidative stress and telomere shortening has been observed in Schwann cells of peripheral neurons in diabetic neuropathy. Since Schwann cells are rich in sorbitol and the conversion of sorbitol to fructose is carried out by sorbitol dehydrogenase, which generates NADH from NAD^+^, an increase in the intracellular concentration of sorbitol can produce osmotic stress. It allows other electrolytes to leak out, affecting the Schwann cells of peripheral neurons and contributing to diabetic neuropathy [181]. In addition, the accumulation of sorbitol and fructose causes a decrease in myo-inositol and taurine concentration, the inhibition of the Na^+^/K^+^-ATP-ase pump, the accumulation of intracellular Na^+^, enlargement of axons, axon-glial cell dysfunction, and a decrease in nerve conduction velocity [182]. Because NADPH is a cofactor in converting sorbitol to fructose, its presence is crucial for forming reduced glutathione (GSH). Without NADPH, GSH content decreases significantly. In this manner, the compromised GSH concentration leads to endothelial damage and the loss of nitric oxide (NO)-mediated vasodilation, well-known factors contributing to telomere shortening [57]. In line with this, the monocytes adhered to the vascular endothelium derived from T2D patients seem to present elevated rates of telomere shortening due to oxidative DNA damage in monocyte precursor cells [113]. In general, endothelial dysfunction is aggravated and leads to significant rates of morbidity and mortality in T2D patients [183]. The capacity of the vascular endothelium to produce NO decreases with age, even in healthy individuals, with no favorable risk factor profile [184].

In chronic uncontrolled hyperglycemia, the hexosamine pathway is also upregulated. In this process, the enzyme glutamine-fructose-6-phosphate-amidotransferase converts fructose-6-phosphate to glucosamine-6-phosphate. Subsequently, glucosamine supports the synthesis of uridine diphosphate-N-acetyl hexosamine (UDP-GlcNAc), which acts as a substrate for N- or O-glycosylation of various proteins. This post-translational alteration is implicated in insulin resistance and the etiology of diabetes and may increase glucotoxicity by decreasing protein function [185]. For example, UDP-GlcNAc enhances O-GlcNAcylation on proteins. This process, therefore, elevates the activity of an important transcription factor, the nuclear factor of activated T-cells (NFAT), which modulates inflammatory pathways and stimulates the expression of pro-inflammatory genes [186]. Moreover, due to the generation of oxidative stress, N-acetylglucosamine (GlcNAc) is responsible for the functional impairment of cells. Overexpression of glutamine-fructo-6-phosphate-amidotransferase increases hydrogen peroxide levels and inhibits the expression of genes for insulin, glucose transporter type 2, and glucokinase. Disproportionate fluctuations in glucose via the hexosamine biosynthesis pathway may induce retinal neuron death, potentially by altering protein glycosylation [187]. Consequently, excessive activation of the hexosamine pathway exacerbates the aging process by generating marked oxidative stress, directly affecting gene expression, and causing genomic instability.

Hence, the PKC pathway can be activated with either fructose-6-phosphate or glyceraldehyde-3-phosphate from continuous glycolysis present in diabetes [188]. Indeed, glycerol-3-phosphate generates diacetylglycerol, which activates several isoforms of PKC. One of the main targets of PKC is NADPH-oxidase, the activation of which increases superoxide anion generation and exacerbates oxidative damage to macromolecules, thereby increasing glucotoxicity [189]. Moreover, PKC activation may enhance oxidative stress via feedback mechanisms. For example, PKCδ induces mitochondrial dysfunction and reactive oxygen species (ROS) production, which can subsequently activate PKC, thereby establishing a cycle of oxidative stress [190]. Under high glucose conditions, the increased diacylglycerol levels promote the activation and synthesis of PKC, which in turn trigger oxidative stress and inflammation, leading to compromised endothelial cell function via NO depletion [191].

**Figure 6:**
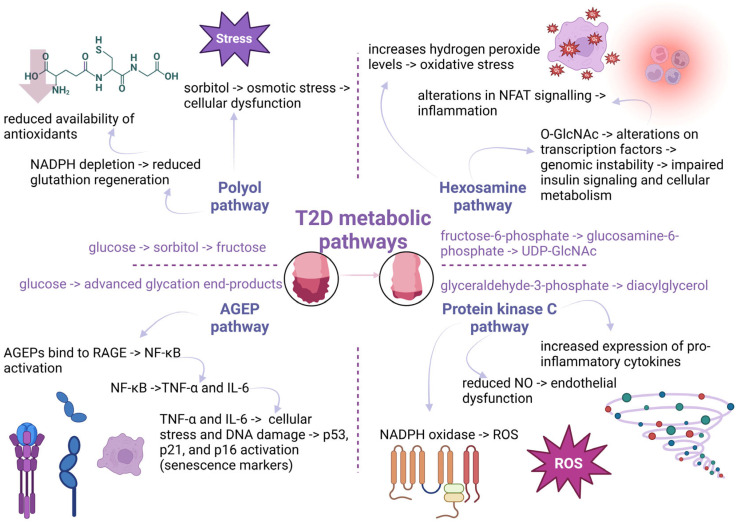
Hyperglycemia-activated metabolic pathways and their by-products contribute to telomere attrition. In type 2 diabetes (T2D), elevated glucose levels activate several harmful metabolic routes. The polyol pathway converts glucose to sorbitol and fructose, causing osmotic stress and cellular dysfunction, while depleting nicotinamide adenine dinucleotide phosphate (NADPH) and reducing glutathione regeneration, weakening antioxidant defenses. The advanced glycation end-product (AGEP) pathway leads to AGE accumulation, which binds to receptors for advanced glycation end-products (RAGE), triggering nuclear factor kappa B (NF-κB) signaling and the release of tumor necrosis factor-alpha (TNF-α) and interleukin-6 (IL-6). These cytokines drive DNA damage and activate senescence markers (p53, p21, p16). The hexosamine pathway, via uridine diphosphate N-acetylglucosamine (UDP-GlcNAc), alters transcription factor activity and insulin signaling, contributing to inflammation and genomic instability. The protein kinase C (PKC) pathway, activated by diacylglycerol from glyceraldehyde-3-phosphate, upregulates pro-inflammatory cytokines and causes endothelial dysfunction through nitric oxide (NO) suppression. All these pathways increase reactive oxygen species (ROS) and promote inflammation, accelerating telomere shortening and cellular aging (created with Biorender.com).

Much research has shown that the contribution of advanced glycosylation end-products (AGEPs) can result in telomere shortening. Indeed, elevated AGEP levels and reduced telomere length have been highlighted, explaining the heightened incidence of major adverse cardiovascular events, particularly in diabetic patients [192]. Another study has illustrated a possible correlation among dietary carbohydrates, AGEPs, and telomere shortening. AGEPs may induce telomere shortening via the generation of pro-inflammatory metabolites, increasing the risk of diabetic complications and age-related diseases during the lifespan [193]. Consequently, the applicability of these biomarkers of glycemic dysfunction in predicting cardiovascular events is suggested. Figure 6 summarizes the most important molecular mechanisms induced by hyperglycemia-activated metabolic pathways contributing to telomere shortening.

In addition, mitochondrial dysfunction can exacerbate oxidative stress and inflammation [194]. It is known that hyperglycemia induces mitochondrial changes, including cytochrome C release, caspase 3 activation, and changes in biogenesis, all leading to programmed cell death [195]. In parallel, glucotoxicity is present along with diminished mitochondrial action potentials and decreased ATP levels. For example, hyperglycemia disturbs neuronal axons because of their high mitochondrial content, defining diabetic neuropathy, which is a condition associated with shorter telomeres [196]. For example, Gordon et al. have identified in a murine model that mitochondrial oxidative stress, increased superoxide levels, and reduced hydrogen peroxide (H_2_O_2_) levels lead to telomere shortening in the airway smooth muscle cells of superoxide dismutase 2 (SOD2)^+/−^ mice. The decrease in telomere length developed in tandem with an increase in telomerase activity and was associated with the emergence of a disease phenotype. The authors proposed that oxidative stress, resulting from an imbalance in mitochondrial reactive oxygen species due to insufficient SOD2 activity, is a model for mitochondrial dysfunction that leads to telomere dysfunction [197].

Furthermore, T2D can perturb telomere dynamics by mediating changes in telomerase activity, as telomerase reverse transcriptase (TERT) promoter polymorphisms have been detected in individuals with T2D [198]. At the cellular level, endothelial cells exposed to high glucose conditions exhibit telomere shortening due to the inhibition of telomerase action [199]. In a clinical setting, the significance of telomerase in T2D is evident in patients with diabetic ulcers, who often exhibit the downregulation of telomerase [9].

In conclusion, the chronic and persistent activation of the metabolic pathways mentioned above (polyol pathway, hexosamine pathway, AGEP pathway, PKC pathway) contributes to the cumulative attrition of telomeres and the generation of a vicious loop characterized by marked oxidative stress and inflammation. These could provide a pertinent explanation for the association between accelerated biological aging and increased mortality in T2D patients. Moreover, understanding the metabolic pathways responsible for the accelerated rate of telomere shortening may offer a novel strategy for developing therapeutic interventions to mitigate specific elements produced by hyperglycemia-activated metabolic pathways that compromise telomere integrity.

Beyond the shared mechanisms of obesity and T2D, telomere shortening has a unique contribution to all metabolic disorders. Telomere shortening leads to cellular senescence in obesity and T2D, as shown in adipose tissue, pancreas, and endothelial cells. In adipocytes, telomere shortening fosters an inflammatory environment that promotes abnormal fat accumulation. In pancreatic cells, telomere shortening reduces the self-renewal capacity of pancreatic cells, preventing insulin secretion. In endothelial cells, telomere shortening is related to the impaired function of blood vessels [56,200]. Telomere shortening can offer insight into monitoring the long-term course of disease and the potential of comorbidities like microvascular and macrovascular side effects. The telomere shortening is often associated with increased prevalence of diabetic neuropathy, retinopathy, and diabetic nephropathy [160].

### 6.2. T2D and Telomere Length in Empirical Studies

Telomere shortening is a characteristic feature of diabetes, independently of type [201]. Mounting evidence from cross-sectional studies has demonstrated the negative effect of diabetes on telomere length, independently of diabetes type [202,203]. In the past, a systematic review has shown that telomere shortening is observed in leukocytes of T1D and T2D patients compared to healthy individuals [204]. The tight link between telomere length and diabetes was directly correlated to an individual’s age, type of diabetes, sex, and BMI [204]. In line with this, several systematic reviews have thoroughly examined the relationship of T2D with telomere length. Initially, a meta-analysis of prospective cohort studies has reported that 44 of 606 healthy participants present higher hazard ratios for the onset of T2D, after monitoring them for 15 years [205]. An additional meta-analysis of 17 cohort studies has also provided insights into the inverse correlation between T2D and telomere length. Indeed, shorter telomere length values are linked to a higher incidence of T2D in an age-dependent manner [204]. In particular, T2D patients younger than 60 showed the most remarkable telomere shortening compared to peers without diabetes or T1D patients [204]. The positive association of telomere shortening with T2D has also been confirmed in two genome-wide association studies (GWAS) [206]. Accordingly, two-sample Mendelian randomization analysis has provided convincing clues that a one-unit genetic decrease in telomere length was linked to a 1.38-fold more significant exacerbation of T2D progression [207].

Furthermore, T2D is a progressive disease accompanied by microvascular and macrovascular complications. Such consequences are diabetic retinopathy, diabetic nephropathy, and diabetic cardiovascular and cerebrovascular diseases [208]. Diabetic retinopathy (DR) is previously thought to be a microvascular illness. Still, it has been recognized as a complex neurovascular disorder affecting the retina’s neural tissue and vascular structure in the past few years [209]. Sharma et al. highlighted the differences in monocytes’ telomere length in different groups composed of individuals with non-proliferative diabetic retinopathy, participants with proliferative diabetic retinopathy, and people with non-insulin-dependent diabetes mellitus without any diabetic retinopathy, and age-matched healthy individuals [210]. As expected, the patients with non-proliferative diabetic retinopathy and participants with proliferative diabetic retinopathy presented significant telomere shortening compared to those with non-insulin-dependent diabetes mellitus without retinopathy [210]. At the same time, healthy controls showed the greatest telomere elongation compared to other groups [210]. In that study, the telomere length was examined through the Southern blot [210]. Different results emerged when telomere length was evaluated through qPCR [211]. The telomeres of patients with proliferative diabetic retinopathy who underwent laser photocoagulation were shown to be longer than those of patients with no diabetic retinopathy or non-proliferative diabetic retinopathy. This difference was statistically significant, with a *p*-value of 0.036 [211].

Diabetic nephropathy (DN) is another common harmful consequence of diabetes, affecting 30% to 40% of diabetic individuals [212]. Hyperglycemia, oxidative burst, impaired insulin signaling, inflammation, hereditary factors, and autoimmune processes are the leading causes of diabetic nephropathy [212]. In males with T2DM who had proteinuria, telomere length was significantly shorter than that of T2D patients without diabetic nephropathy [213].

After analyzing men with T2D, an inverse relationship was found between telomere length, glucose concentrations, and levels [164]. In this direction, in vitro evidence has demonstrated a positive association between telomere shortening and hyperglycemia [214]. In this context, telomerase overexpression can reverse hyperglycemia-mediated senescence [214]. In the clinical setting, a randomized controlled trial (RCT) supported the idea that individuals with T2D showed an accelerated telomere length in those with atherosclerotic plaques compared to those without them [215]. Interestingly, metformin attenuated the involved molecular pathways underlying longevity in people with prediabetes in other RCTs [216].

Regarding the prognostic value of telomere shortening in the context of T2D, several prospective studies have demonstrated that telomere shortening can serve as a predictive biomarker for monitoring the onset of T2DM [205,217]. In particular, Zhao et al. have highlighted that 12.5% of participants demonstrated a higher possibility of diabetes during an average of 5.5 years of follow-up [218]. Numerous studies have examined the association between telomere length and the development of T2D, showing that longer telomeres have a protective role for T2D, whereas shorter telomeres than the average confer a higher risk of T2D. In particular, a 6-year follow-up study comprising 82,069 postmenopausal women and their respective control individuals provided meaningful information about the risk of diabetes based on the analysis of telomere dynamics. The study has supported the finding that a 6% reduction in the risk of T2D is associated with every one kilobase (kb) increase in telomere length, with the relationship varying depending on ethnicity [219]. Moreover, telomere length was measured in 12,792 Mexican Americans, including 533 individuals with T2D. A 48% reduction in the probability of developing T2D pathology was associated with telomere length elongation [220]. Accordingly, the Danish Twin Registry discovered a correlation between shorter baseline telomere length and a higher likelihood of insulin resistance worsening over 12 years [207].

To sum up, telomere length serves as a marker for increasing our understanding of the pathogenesis of obesity or T2D. Because telomere length varies among obese individuals, it is not currently used as a diagnostic marker to identify obesity or T2D. In examining the diagnostic use of telomere length in T2D, a Mendelian randomization study has highlighted that each reduction in telomere length in obese individuals increases their susceptibility to glycemic progression, requiring insulin treatment [207]. Since obesity is closely linked to metabolic syndrome, a Mendelian randomization study has shown that longer telomere length values confer an increased risk of developing metabolic syndrome [221].

When T2D is present with other comorbidities, a recent RCT has shown that telomere shortening can serve as a predictive biomarker for the onset of T2D, as coronary heart disease patients with short telomeres have a higher risk of developing T2D [200]. The Mediterranean diet was presented as a diet that reduces the incidence of T2DM in patients with coronary heart disease [200]. As a result, telomere length was inversely associated with the risk of diabetes. Table 2 summarizes the conclusive empirical studies that have linked type 2 diabetes (T2D) and telomere length.

Therefore, telomere length should be considered a reliable biomarker for predicting the probability of diabetes onset and a biomarker of the disease state. Thus, routine determinations of telomere length should be employed in the context of the ever-evolving personalized therapy.

## 7. Limitations

Several limitations prevent the use of telomere length in the context of metabolic disorders, including obesity and T2D. First of all, telomere length values are different in each tissue of every individual, especially in an individual with a metabolic disorder [225]. Apart from the differences in telomere length values among tissues, different individuals and even the same person can display different telomere length values over time, reflecting the progression of the disease. Secondly, there are various methodologies to evaluate telomere length values like quantitative polymerase chain reaction (qPCR), terminal restriction fragment (TRF) analysis, quantitative fluorescence in situ hybridization (qFISH), Southern blot etc. [8]. Thirdly, it is unclear whether telomere shortening is a driving force behind metabolic disorders or a consequence of them.

## 8. Discussion

During the last decades, the prevalence of obesity has increased. Obesity is characterized by the progressive accumulation of fat in the body, accompanied by an increased proinflammatory response and oxidative stress, which compromises insulin sensitivity. In parallel, there has been significant progress in the impact of obesity and T2D on aging, negatively affecting telomere length values. The rate of telomere shortening has been observed to be more pronounced in T2D conditions than that reported in T1D conditions. At a molecular level, oxidative stress and inflammatory responses are fundamental parameters of metabolic disorders, including obesity and T2D, that drive telomere dysfunction. These factors of metabolic disturbance propagate DNA damage, accelerating telomere shortening.

Although telomere length is not yet a direct tool for optimizing therapeutic strategies, it is being explored more extensively as a potential therapeutic target in obesity and type 2 diabetes (T2D), to monitor either disease progression or the effect of various therapeutic options. In obesity, telomere length can serve as a therapeutic target to assess the effectiveness of various anti-obesity treatment options, including exercise, diet, and bariatric surgery [133]. The interaction between the immune system and redox balance is crucial for maintaining metabolic health throughout an individual’s life [226]. Over the last few years, a growing interest in the antioxidants and anti-inflammatory factors that alleviate metabolic dysfunction and telomere shortening has emerged. In this regard, antioxidant and anti-inflammatory agents can mitigate the onset and progression of obesity, T2DM, and metabolic syndrome by affecting telomere length dynamics. For example, anti-inflammatory agents and antioxidants (vitamins C, E, and polyphenols) can maintain telomere length values, preventing obesity and its consequences [133]. In particular, a Mediterranean diet or regular exercise can help sustain telomere length dynamics, thereby alleviating the complications of obesity [152]. Notably, omega-3 polyunsaturated fatty acids (n-3 PUFAs) and polyphenols are enriched in the Mediterranean diet, which helps prevent oxidative stress and maintain telomere length [133,227,228]. Following a 5-year Mediterranean diet, participants in the PREDIMED randomized controlled trial had longer telomeres [152]. The patients with the longest telomeres experienced an even greater decrease in adiposity parameters during the study [152]. In T2D, telomere length is a therapeutic target to monitor the efficacy of several anti-diabetic options like metformin [216]. Indeed, metformin can exert its beneficial effect on T2D by reducing senescence and telomere shortening [216]. According to new research, sodium-glucose cotransporter 2 (SGLT2) inhibitors and glucagon-like peptide 1 (GLP-1) receptor agonists may reduce overall oxidative stress, thereby contributing to the maintenance of telomere length [229,230]. Regarding telomerase activators, such as GRN510 and TA-65, which extend telomere length, studies have been conducted in vitro and in vivo settings; however, their clinical use in metabolic disorders remains absent. Several examples have shown that telomerase activators can ameliorate metabolic function, alleviating the pathogenesis of metabolic disorders [231]. For example, there are therapeutic strategies that increase telomerase activity, delaying telomere shortening. For example, statins increase telomerase activity, which reduces inflammation and ultimately mitigates telomere shortening [232]. Senolytics is another therapeutic strategy that eliminates senescent cells, which are characterized by abnormally short telomeres. Senolytics can enhance and normalize disturbed metabolism. For example, senolytics like quercetin can help maintain telomere length, as shown in a randomized controlled study of 88 participants [233]. To sum up, there are several approaches that exert their therapeutic benefit targeting telomere length dynamics in metabolic disorders, and still other are under research to compromise the pathology of metabolic disorders.

## Figures and Tables

**Table 1:** Obesity and telomere length dynamics in murine models of disease.

Sample	Effect	Method	Conclusion	Reference
Mice, embryos, oocytes	Negative (in obese mice, telomere shortening was observed in oocytes and early embryos)	Quantification of the TRF1 shelterin protein and γ-H2AX foci, using immunofluorescence staining	Maternal obesity negatively affects the quality of oocytes and embryo development through telomere disruption.	[156]
Mice	Negative (the HFD-treated young mice presented increased TRF2 gene expression by 47% and 80% in aged mice, 16 months; regardless of diet, POT1b expression was upregulated in 16-month-old mice by 35–60%)	qPCR	Age and obesity affected the gene expression of telomerase and the shelterin protein complex in adipose tissue.	[117]
Mice	Negative (HFD-treated mice showed telomere shortening in the testis and increased expression of senescent makers through the endoplasmic reticulum stress)	qPCR for evaluation of telomere length, Western blot, and immunofluorescent stainings for senescent markers	HFD-treated male mice experienced endoplasmic reticulum stress, causing the senescence of their testicles.	[157]

Abbreviations: HFD: high-fat diet; qPCR: quantitative polymerase chain reaction.

**Table 2:** T2D and telomere length in empirical studies.

Experimental Group	TL Assessment Method	Conclusions	Reference
1002 patients (20–75 years old) with CHD—502 patients on a Mediterranean diet and 500 on a low-fat diet, out of which 462 did not have T2M at baseline	qPCR	T2D risk is positively correlated with shorter telomeres in CHD patients; the Mediterranean diet was more effective in reducing the risk of T2D among patients with shorter telomeres.	[222]
742 T2D patients (Caucasians, South Asians, Afro-Caribbeans); 81 young healthy controls; 367 older healthy controls	qPCR	T2D patients had shorter telomeres; UCP2-866A allele patients (linked to higher oxidative stress) presented with shorter telomere length.	[114]
5506 Chinese patients with T2D	qPCR	Shorter telomeres were positively correlated with faster glycemic progression, independent of risk factors; genetically determined shorter telomeres were linked to a higher risk of T2D progression; telomere length might be a predictive biomarker for T2D.	[207]
183 newly diagnosed T2D patients	qPCR	Longer telomere length values at baseline are predictive of higher T2D remission probability; responders to the Mediterranean diet or low-fat diet showed increased telomerase activity and fewer shortened telomeres than non-responders; T2D patients with a poorer profile of insulin resistance or beta-cell functioning had significant telomere shortening.	[200]
130 males with IHD (65 with T2M; 65 without T2M)	qPCR	T2D and IHD had shorter telomere length values; telomere shortening could be a biomarker for T2D in IHD patients.	[223]
1354 people (682 T2D patients; 672 controls)	genotyping of telomere-related gene variants linked to T2D	Certain genetic variants (rs9419958, rs4783704, rs16847897, rs10936599, and rs74019828) were associated with a higher risk of T2D; telomere maintenance genes could contribute to T2D risk.	[224]

Abbreviations: CHD: coronary heart disease; T2D: type 2 diabetes; qPCR: quantitative polymerase chain reaction; IHD: ischemic heart disease.

## Abbreviations

(T2D)	type 2 diabetes mellitus
(TRF1)	telomeric repeat-binding factor 1
(TRF2)	telomeric repeat-binding factor 2
(NEFAs)	non-esterified fatty acids
(DALYs)	disability-adjusted life years
(CVD)	cardiovascular disease
(BMI)	body mass index
(WHO)	World Health Organization
(TNF-α)	tumor necrosis factor-α
(IL-6)	interleukin-6
(MCP-1)	monocyte chemoattractant protein-1
(HIF-1α)	hypoxia-inducible factor-1α
(NF-κB)	nuclear factor-κB
(JNK)	c-Jun amino-terminal kinase
(PKC)	protein kinase C
(GLUT4)	glucose transporter type-4
(SASP)	senescence-associated secreted proteome
(TG)	triglyceride
(PPAR-γ)	peroxisome proliferator-activated receptor-γ
(LPL)	lipoprotein lipases
(FFA)	free fatty acids
(AMPK)	monophosphate-activated protein kinase
(T1D)	type 1 diabetes mellitus
(NO)	nitric oxide
(eNOS)	endothelial nitric oxide synthase
(GFs)	growth factors
(ROS)	reactive oxygen species
(UDP-GlcNAc)	uridine 5-diphosphate-N-acetylglucosamine
(TCA)	tricarboxylic acid
(ETC)	electron transport chain
(acetyl-CoA)	acetyl coenzyme A
(TXA2)	thromboxane A2
(COX-2)	cyclooxygenase-2
FDR	first-degree relatives
(PAI-1)	plasminogen activator inhibitor-1
(C/EBP-β)	CCAAT/enhancer-binding protein-β
(RNS)	reactive nitrogen species
(O2-)	superoxide anion
(ULK-1 and ULK-2)	unc-51-like kinase 1/2
(ATP)	adenosine triphosphate
(8-epi-PGF2α)	8-epi-prostaglandin F2α
(CRP)	C-reactive protein
(EPCs)	endothelial progenitor cells
(CAD)	coronary artery disease
(MAPK)	mitogen-activated protein kinase
(mTOR)	mammalian target of rapamycin
(NADPH)	ninicotinamide adenine dinucleotide phosphate
(bps)	base pairs
(IMT)	intima-media thickness
(LDL)	low-density lipoprotein
(MDA)	malondialdehyde
(UCP2)	uncoupling protein 2
(TGF-β)	transforming growth factor-β
(IGF-1)	insulin-like growth factor 1
(PGE2)	prostaglandin E_2_
(RAGE)	glycation end-product receptor
(H202)	hydrogen peroxide
(SOD2)	superoxide dismutase 2
(TERT)	telomerase reverse transcriptase
(GSH)	glutathione
(qPCR)	quantitative polymerase chain reaction;
(UDP-GlcNAc)	uridine diphosphate-N-acetyl hexosamine
(IHD)	ischemic heart disease
(CHD)	coronary heart disease
(GWAS)	genome-wide association studies
(DR)	Diabetic retinopathy
(DN)	Diabetic nephropathy
(RCT)	randomized controlled trial
(kb)	kilobase
(qFISH)	quantitative fluoresce in situ hybridization
